# Fiber Optic Sensor for Acoustic Detection of Partial Discharges in Oil-Paper Insulated Electrical Systems

**DOI:** 10.3390/s120404793

**Published:** 2012-04-12

**Authors:** Julio Posada-Roman, Jose A. Garcia-Souto, Jesus Rubio-Serrano

**Affiliations:** Department of Electronics Technology, Optoelectronics and Laser Technology Group, Universidad Carlos III de Madrid, Av. Universidad 30, E-28911 Leganés, Madrid, Spain; E-Mails: jsouto@ing.uc3m.es (J.A.G.-S.); jrserran@ing.uc3m.es (J.R.-S.)

**Keywords:** optical fiber sensors, interferometry, acoustic emission, ultrasounds, partial discharges, transformers

## Abstract

A fiber optic interferometric sensor with an intrinsic transducer along a length of the fiber is presented for ultrasound measurements of the acoustic emission from partial discharges inside oil-filled power apparatus. The sensor is designed for high sensitivity measurements in a harsh electromagnetic field environment, with wide temperature changes and immersion in oil. It allows enough sensitivity for the application, for which the acoustic pressure is in the range of units of Pa at a frequency of 150 kHz. In addition, the accessibility to the sensing region is guaranteed by immune fiber-optic cables and the optical phase sensor output. The sensor design is a compact and rugged coil of fiber. In addition to a complete calibration, the *in-situ* results show that two types of partial discharges are measured through their acoustic emissions with the sensor immersed in oil.

## 1. Introduction

Oil-paper insulation systems are commonly used in power apparatus such as power transformers and high voltage cables, among others. In power transformers the insulation system is the most important part of the internal insulation and it largely determines its operational reliability. Thus, condition assessment of the insulation system in a timely manner, ensures reliable operation of the transformer, and maximizes equipment utilization [1].

Partial discharges (PD) are a cause and a symptom of the degradation of the insulation system and its activity monitoring is used as a tool for insulation condition assessment in power transformers [2]. PD are small electrical sparks present in an insulator as result of the electrical breakdown of a gas (for example air) contained within a void or in a highly non-uniform electric field [3]. The sudden release of energy caused when a PD occurs produces a number of effects like chemical and structural changes in the materials, electromagnetic signal generation and acoustic emissions (AE) [4]. These induced effects are used for its detection. Techniques such as dissolved gas analysis (DGA) electrical measurements of high frequency transients (HF-VHF), detection of electromagnetic signals generated in the UHF band and ultrasound AE detection are used for this propose. Among these techniques AE ultrasound detection offers great advantages such as the possibility of on-line testing and the ability to locate where PD activity is occurring, which is helpful in large test objects like power transformers.

The UHF technique has been applied to transformers for the detection and location of PD and show promising results [5,6]. However, the installation of the sensors is a drawback because they require an electromagnetic wave “view” into the tank, so the installation of a dielectric window that provides a mounting point for the sensors on the transformer tank is needed. Moreover, electromagnetic waves from PD are affected by reflections and refractions produced by obstacles such as the core, copper conductors, *etc.* producing multiple paths before arriving to the sensors, which complicates the localization of the source.

The detection and location of PD using AE techniques are commonly done with external piezoelectric (PZT) ultrasound sensors mounted on the tank wall, which have narrowband detection at 150 kHz. However, they suffer the same problem as UHF detection related to a multi-path signal and, in addition, to a low level detected signal as a consequence of the attenuation of the acoustic waves through the oil. Therefore, it is desirable to have sensors that can be placed inside the transformer, close to the PD, that overcome these problems.

Recently several fiber optic (FO) sensors of different types have been developed for PD detection within power transformers, such as those based on FO Fabry-Perot cavities with resonant response at around 150-kHz [7–9], or the ones based on Fiber Bragg Gratings [10]. However, the sensitivity of these sensors is moderate and they show a great dependence on the technology of integration.

Another PD sensing approach for internal installation is based on the interferometric measurement of AE with a single-mode optical fiber as the intrinsic transducer of ultrasounds into optical phase [11,12]. More sensitivity can be achieved with this technique by using long fibers in the sensing arm but the frequency response would be a drawback.

This work is focused on the development, characterization and testing of a fiber optic intrinsic sensor for acoustic detection of PD. It is organized as follows: Section 2 is devoted to the principle of sensing of the FO sensor and the description of the read-out system. The calibration of the sensor is presented in Section 3. Several tests under real conditions are presented in Section 4. These were carried out in a high voltage laboratory where the sensor was tested with PD generated in transformer oil. Finally, the conclusions are included in Section 5.

## 2. Principle of Sensing and Interferometric Read-Out System

### 2.1. Sensing Principle

Acoustic emissions are pressure variations in an elastic medium. The principle of sensing of an intrinsic fiber optic acoustic sensor is based on the change in the optical path length produced by the strain induced by the acoustic pressure waves. For an interferometric approach, where the optical phase of the interfering light contains the information of the measured magnitude, the phase of the light (*Φ*) passing through a piece of optical fiber of longitude *L* is given by:

(1) $$\phi =\beta L=\frac{2\pi \eta_{\text{eff}}}{\lambda }L$$

where *β* is the propagation constant, *η_eff_* is the effective refractive index of the fiber and λ is the optical wavelength. The change in the phase is then:

(2) $$\begin{array}{cc} \Delta \phi & =\beta \Delta L+L\Delta \beta \\ & =\Delta \phi_{1}+\Delta \phi_{2} \end{array}$$

The first term in the last expression represents the phase shift due to the axial stretching of the fiber:

(3) $$\Delta \phi_{1}=-\frac{\beta L}{E}(1-2\upsilon )\Delta P$$

where *ν* is the Poisson ratio, *E* is the Young modulus and ΔP is the acoustic pressure change.

The second term in Equation (2) is the change of the propagation constant which depends on the change of the refractive index (strain-optic effect) and the fiber diameter produced by the strain. However, the effect of the change on diameter is proved to be negligible [13] so Δ*Φ*_2_ can be written as:

(4) $$\Delta \phi_{2}=\frac{\beta Ln^{2}}{2E}\left(1-2\upsilon \right)\left(p_{11}+2p_{12}\right)\Delta P$$

where *p*_11_ and *p*_12_ are elements of the strain-optic tensor. Substituting Equations (3) and (4) in Equation (2) it is obtained:

(5) $$\frac{\Delta \phi }{\phi \Delta P}=\frac{\left(1-2\upsilon \right)}{E}\left[\frac{n^{2}}{2}\left(p_{11}+2p_{12}\right)-1\right]$$

the expression Δ*Φ*/(*Φ*Δ*P*) is known as the normalized acoustic phase responsivity (NR) and it is expressed in Pa^−1^.

### 2.2. Design of the Sensor Probe

Some specific characteristics of the external ultrasound sensors that are commonly used for PD detection have been taken for the development of the proposed fiber optic sensor: sensitivity of 10 mV/Pa at the frequency of 150 kHz and bandwidth between 100 kHz and 300 kHz [14]. Moreover, PD acoustic emissions are expected in a typical range of 1 Pa to 10 kPa. This makes the interferometric measurement suitable for the application due to the high range-resolution that it is able to achieve. In this case, the most important parameter of design is the resolution (1 Pa). The proposed sensitivity is like that of an R15i sensor (10 mV/Pa) and corresponds to −180 dB re-rad-μPa^−1^ (1 × 10^−9^ rad/μPa). Under these conditions and assuming a quasi-linear output range of 20 V in the interferometer (stabilized for homodyne detection), the input dynamic range in the system is 2 kPa. These requirements should be appropriate and they are the initial requirements of design.

The sensor design is based on a fiber optic coil in a multilayer configuration which will be exposed to AE. Since the phase sensitivity is inversely proportional to *λ*, as can be seen in Equation (5), a short optical wavelength (633 nm) single-mode fiber is selected for the construction of the sensor in order to obtain higher phase sensitivity. The fiber used to build the sensor is the model: SCSM-633-HP1, which is a coated fiber with an operating wavelength range of 600–760-nm. A simple calculation of the fiber length that is needed to obtain the desired sensitivity is done using the NR for a typical coated fiber, which is ≈−330-dB-re-μPa^−1^ [15], and the optical wavelength of 633-nm aforementioned for the interrogation of the sensor. With these parameters the total optical phase (Equation (1)) in 1-m of fiber corresponds to 143-dB-re-rad, therefore the sensitivity of the sensor will be −187 dB re-rad-μPa^−1^ m^−1^. It means that 2-m of fiber is needed to obtain the desired sensitivity.

**Figure 1.**
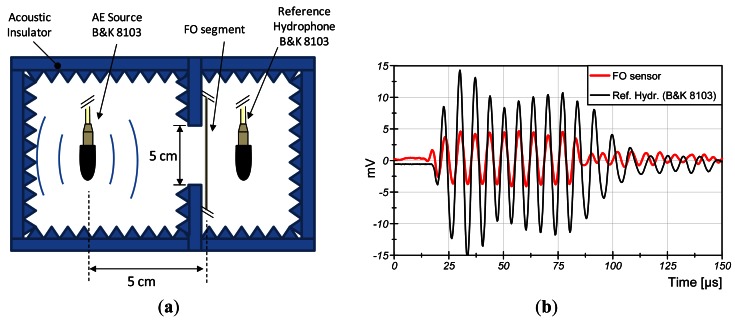
(**a**) Experimental set-up for the measurement of fiber sensitivity; (**b**) The same AE detected with the FO segment and the calibrated hydrophone.

Since the value of NR that is used for the calculation of the sensor fiber length is an approximated value, an experimental measurement was carried out in order to obtain the real value of the responsivity for this fiber. The set up for the test is shown in Figure 1.

In this experiment a FO segment of 5-cm is immersed in water and a calibrated hydrophone (B&K 8103) is placed at the same distance to the source. This is observed in Figure 1(b): both signals start at the same time. Both are exposed to the same AE of 150-kHz emitted with a transducer placed at 5-cm. Figure 1(a)—the reference hydrophone and the FO segment are separated for clarity. The segment of fiber is interrogated by a Mach-Zhender interferometer with an output range of 12-V. The reference hydrophone has a sensitivity of 200 μV/Pa@150 kHz.

The results of the measured sensitivity are shown in Figure 1b. The AE of 100-Pa is observed in the fiber segment as 1.45 × 10^−3^-rad. This result shows a responsivity of −190 dB re rad-μPa^−1^·m^−1^. Therefore, the length of fiber needed for the sensor is 3.2-m.

**Figure 2.**
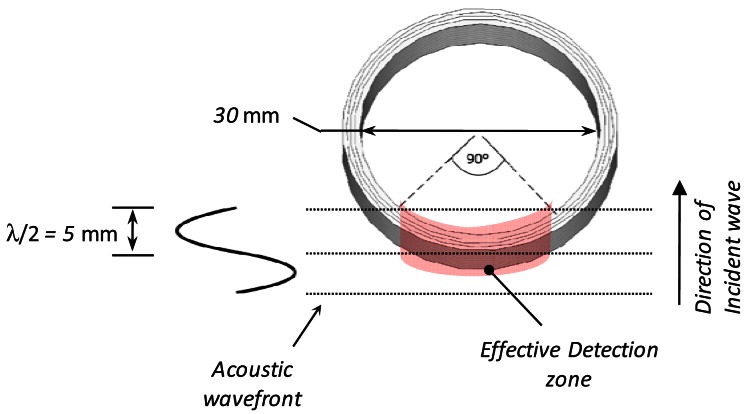
Detail of the effective sensitive zone of the sensor at the desired frequency.

Before the construction of the sensor, a design problem related with the acoustic frequency of interest (150 kHz) and the dimensions of the sensor has to be analyzed. When an acoustic wave of 150 kHz is propagating in the transformer oil (velocity of 1,400 m/s) the acoustic wavelength is ∼10 mm. The minimum diameter achievable in the coil of conventional fiber is around 30-mm in order to avoid excessive optical losses. Therefore, since the dimensions of the sensor are greater than the desired wavelength, a phase difference of the acoustic field is present across the coil and this causes a reduction of the average pressure onto the sensor. This effect reduces the sensitive zone of the sensor that brings a net contribution free of the averaging effect and it is about 1/4 of the total fiber length of the coil (Figure 2). As a consequence, a fiber length of at least 13 m is needed for the building of the sensor.

The sensor was made by winding multiple layers of fiber around a former of 30 mm in diameter. The fiber in the coil was 17-m long. This amount of fiber is arranged in five layers of 5 mm of width.

### 2.3. Interferometric Read-Out System and Electronic Conditioning

**Figure 3.**
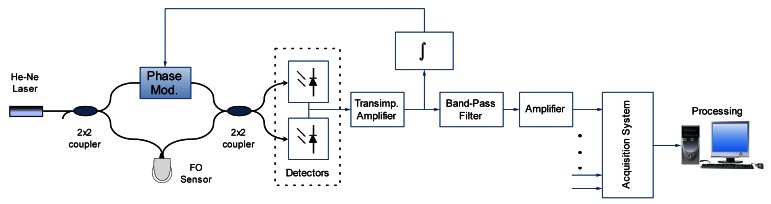
Diagram of the optoelectronic instrumentation system.

In order to prove the sensor, a scheme based on an all-fiber Mach-Zehnder interferometer with homodyne demodulation was used. The block diagram of the implemented optoelectronic instrumentation system is shown in Figure 3.

In this scheme a He-Ne (633 nm) laser coherent light source is used to interrogate the sensor. The detection of the optical phase encoded in the intensity output is done through two balanced photodetectors. Differential transimpedance amplification is used to compensate the effect of the mean optical power onto each photodetector in order to improve the performance with a DC null configuration. A homodyne demodulation is used to set the interferometer operating point at the maximum sensitivity and in the middle of the quasi-linear output range (−π/4 rad to π/4 rad). It is done by a feedback loop that integrates the error signal and actuates through a phase modulator which is connected to the fiber reference arm to compensate the temperature drift and other low frequency disturbances on the interferometer. The phase modulator implemented here is able to compensate up to 50π rad at frequencies below 200 Hz. Moreover, a control of the optic polarization has been included in order to avoid the interference signal fading.

Once the optical phase is converted into a voltage signal, it is conditioned through a band-pass filtering stage with a resonance frequency of 150 kHz in order to adjust the bandwidth of the FO sensor to the acoustic PD emissions. It equalizes the frequency response obtained with the sensing probe, as well as it attenuates the disturbances that are produced by other acoustic sources in the transformers (such as the Barkhausen noise [14]).

The stabilized interferometer has a voltage output proportional to the optical phase change given by:

(6) $$V_{s}=2I_{0}\Delta \phi R\eta G_{T}G_{F}$$

where *I*_0_ is the mean optical power at each photo-detector, *R* is the responsivity of the photo-detectors, *η* is a factor between 0 and 1 that is determined by the contrast of the interference (in this case 0.6). G_T_ and G_F_ are the transimpedance gain and the band-pass filter gain respectively.

## 3. Calibration of the Sensor

**Figure 4.**
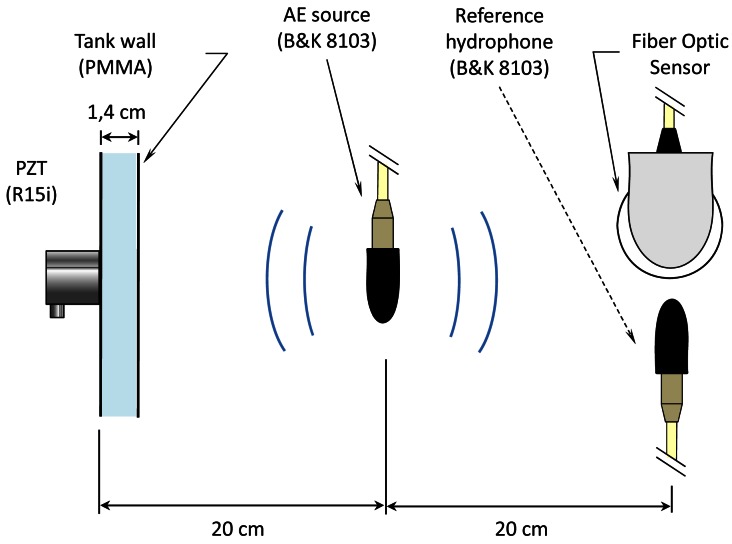
Experimental set-up for the calibration of the sensor in water.

The calibration of the sensor was carried out in an acoustic test bench in water (Figure 4). In this scenario the AE produced by PD can be reproduced, but without high voltage elements present in the set-up. This provides a controlled and safety environment for the calibration. The test bench includes an immersed calibrated hydrophone (Brüel & Kjaër 8103) and an external sensor (model: R15i-AST), which is commonly used in acoustic PD detection, mounted on the wall of the tank. The generation of the acoustic emission is done by using a transducer identical to the reference hydrophone.

### 3.1. Frequency Response of the Sensor and Sensitivity at 150 kHz

**Figure 5.**
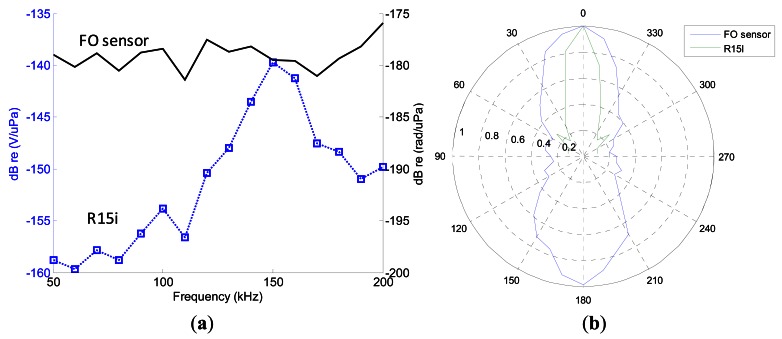
(**a**) Frequency response of the FO sensor; (**b**) Comparative analysis of the directivity of the external sensor and the fiber optic internal sensor at 150-kHz.

A comparative analysis of the frequency response between the internal FO sensor and the external R15i sensor was done in order to evaluate if the bandwidth of the fiber optic sensor is suitable for the application. In this test, a frequency sweep was applied from 50 kHz to 200 kHz: in the range of frequencies in which the hydrophone is calibrated. The results are shown in Figure 5(a).

The sensitivity obtained for the FO sensor at 150-kHz was 1.1-mrad/Pa (11 mV/Pa for 20-V output range in the interferometer). Moreover it has broader bandwidth (100 kHz–300 kHz were observed with a flat response). This is useful for PD identification because part of the emitted acoustic energy is found in that portion of the spectrum. The resultant noise equivalent bandwidth of the sensor is a drawback in this case.

### 3.2. Directionality

The directional characteristics were characterized at 150-kHz and the results are shown in Figure 5(b). A wider detection field was found in the FO sensor. The directivity span is approximately ±30° compared to ±15° of the external sensor R15i. This result shows an advantage for PD detection with FO sensors over conventional external detection because wide zones should be inspected in large transformers.

## 4. Testing the Fiber Optic Sensor with PD Generated in Transformer Oil

**Figure 6.**
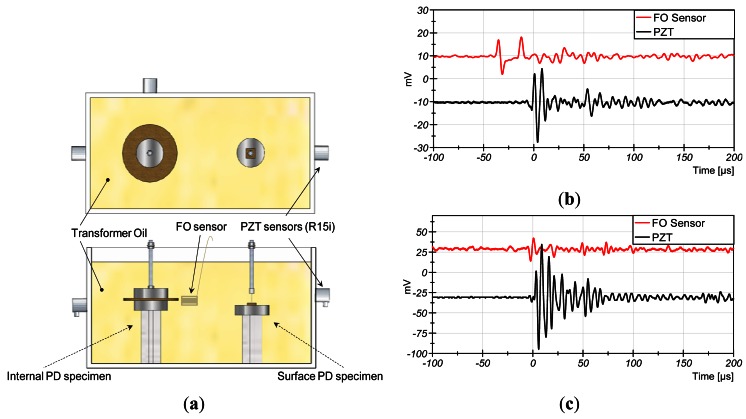
(**a**) High-voltage test bench of PD generation in oil; (**b**) Average of eight signals detected for internal PD; (**c**) Signals detected for surface PD.

A four layer sensor was manufactured and tested with real PD generated in transformer oil in order to test its performance in real conditions. In this test bench PD are generated with high voltage electrodes immersed in oil. Two different types were generated in order to produce representative PD: plane-plane electrodes for internal PD and needle-plane electrodes for surface PD (Figure 6(a)).

The set-up was configured to detect with two sensors, internal (FO sensor) and external (R15i), at the same time. The FO sensor was located at 9-cm and 15-cm from the internal PD source and the surface PD source, respectively. The external sensor was located at 15 cm from the source in both cases.

The results of the tests are shown in Figure 6(b,c). The FO sensor shows suitable sensitivity to detect the acoustic emission of PD in both cases. The amplitude of the detected acoustic emissions is about ∼1.3 Pa. Different waveforms are detected by the two sensors because their different frequency response. The broader bandwidth of the FO sensor produces shorter transient response than the PZT.

In addition, two peaks are detected by the FO sensor, which are delayed 23 μs. In this experiment, the acoustic absorber is removed from the middle of the coil. Therefore, the AE is detected by the first sensitive face and it travels to the second sensitive face where is also detected with a delay.

Based on previous works, if bare fiber is exposed for long time periods to the conditions inside transformers, it becomes tinted with the color of the oil. However, optical fibers withstand harsh environments and do not show permanent changes of the optical transmission and of the elasto-optic properties [16–18]. Hard coatings such as Teflon give better acoustic sensitivity, flat frequency response and thermal drift desensitization. Soft coatings such as acrylate based elastomers have narrower band-pass sensitivity to ultrasounds, which is desirable in transformers.

## 5. Conclusions

A fiber optic sensor for PD detection in transformer oil has been developed. It has been characterized in an acoustic test bench in water and also tested with real PD generated in oil. In the characterization a sensitivity of 1.1 m rad/Pa at 150-kHz (11 mV/Pa with 20 V of range at interferometer output) and a wider detection field (±30°) compared with an external sensor (±15°) has been shown. The test of the sensor with real PD shows that it has suitable sensitivity and enough resolution to detect acoustic pressure as low as 1.3 Pa.

It is a cost-effective solution compared with other fiber optic sensors which can also be immersed in oil. It offers the possibility of a multichannel configuration for an easy deployment of several sensors to locate the source of partial discharges through the time of flight of the acoustic emissions.

The optoelectronic scheme with homodyne demodulation that is used for the characterization of the FO sensor is practical for one channel application. However, a new optical interrogation scheme is needed for the implementation of multiple internal sensors (at least four) to locate the PD source. A heterodyne interferometer is proposed, which is able to drive multiple channels. With this technique, measurements with improved signal to noise ratio are expected because the measuring signal modulates a high frequency carrier.

Further work will be focused on the stability of the optical fiber sensor head and the AE sensitivity drift in controlled conditions of oil immersion and accelerated aging that emulates the exposition to the transformer environment for long time periods.
